# Novel missense mutation of SASH1 in a Chinese family with dyschromatosis universalis hereditaria

**DOI:** 10.1186/s12920-021-01014-w

**Published:** 2021-06-26

**Authors:** Lu Cao, Ruixue Zhang, Liang Yong, Shirui Chen, Hui Zhang, Weiwei Chen, Qiongqiong Xu, Huiyao Ge, Yiwen Mao, Qi Zhen, Yafen Yu, Xia Hu, Liangdan Sun

**Affiliations:** 1 Department of Dermatology, the First Affiliated Hospital of Anhui Medical University, Hefei, China; 2 Institute of Dermatology, Anhui Medical University, Hefei, China; 3 Key Laboratory of Dermatology, Anhui Medical University, Ministry of Education, Hefei, China; 4 Inflammation and Immune Mediated Diseases Laboratory of Anhui Province, Hefei, China; 5 Anhui Provincial Institute of Translational Medicine, Hefei, China

**Keywords:** *ABCB6* gene, Dyschromatosis universalis hereditaria, Mutation, *SASH1* gene

## Abstract

**Background:**

Dyschromatosis universalis hereditaria (DUH) is a pigmentary dermatosis characterized by generalized mottled macules with hypopigmention and hyperpigmention. *ABCB6* and *SASH1* are recently reported pathogenic genes related to DUH, and the aim of this study was to identify the causative mutations in a Chinese family with DUH.

**Methods:**

Sanger sequencing was performed to investigate the clinical manifestation and molecular genetic basis of these familial cases of DUH, bioinformatics tools and multiple sequence alignment were used to analyse the pathogenicity of mutations.

**Results:**

A novel missense mutation, c.1529G>A, in the *SASH1* gene was identified, and this mutation was not found in the National Center for Biotechnology Information Database of Short Genetic Variation, Online Mendelian Inheritance in Man, ClinVar, or 1000 Genomes Project databases. All in silico predictors suggested that the observed substitution mutation was deleterious. Furthermore, multiple sequence alignment of *SASH1* revealed that the p.S510N mutation was highly conserved during evolution. In addition, we reviewed the previously reported DUH-related gene mutations in *SASH1* and *ABCB6*.

**Conclusion:**

Although the affected family members had identical mutations, differences in the clinical manifestations of these family members were observed, which reveals the complexity of the phenotype-influencing factors in DUH. Our findings reveal the mutation responsible for DUH in this family and broaden the mutational spectrum of the *SASH1* gene.

**Supplementary Information:**

The online version contains supplementary material available at 10.1186/s12920-021-01014-w.

## Background

Dyschromatosis universalis hereditaria (DUH) is an infrequent hereditary dermatosis accompanied by generalized mottled macules with hypopigmention and hyperpigmention. This genodermatosis usually begins at birth or in early childhood and affects almost the whole body; nails, hair, and teeth can even be involved. Some cases may be accompanied by systemic damage, such as deafness, visual impairment, and neurological symptoms.

DUH was first reported by Ichikawa and Hiraga in 1933. As a hereditary disorder, determining the causative gene is particularly important for the diagnosis and treatment of DUH. As early as 2003, Chinese scholars performed genome-wide screening in two DUH families from Henan and Yunnan for linkage analysis and confirmed that the gene associated with dominant dyschromatosis symmetrica hereditaria (DSH) maps to chromosome 6q24.2-q25.2 [1]. It is worth noting that Xing et al. diagnosed the family with DSH, while the results of subsequent studies suggested that the family should actually have been diagnosed with DUH [2]. In 2008, Stuhrmann et al. [3] discovered an autosomal-recessive inheritance associated with DUH region 12q21-q23 in the Arab population. In 2013, Zhang et al. identified a third DUH-related pathogenic region, 2q33.3-q36.13, and confirmed that ACBC6, located at 2q35, is the pathogenic gene of DUH [4].

Therefore, DUH can be divided into three types, DUH1 (Online Mendelian Inheritance in Man (OMIM) 127500), DUH2 (OMIM 612715) and DUH3 (OMIM 615402), based on the different linkage regions located in the 6q24.2-q25.2, 12q21q23 and 2q35 regions, respectively. DUH1 and DUH3 are inherited in an autosomal-dominant manner, while DUH2 is inherited in an autosomal-recessive manner. *ABCB6* and *SASH1* [5] are recently reported pathogenic genes related to DUH. We collected data from a Chinese family with DUH with a novel missense mutation in *SASH1* and reviewed published literature on mutations in SASH1 related to pigmentation abnormalities.

## Methods

### Clinical findings and diagnosis

**Fig. 1:**
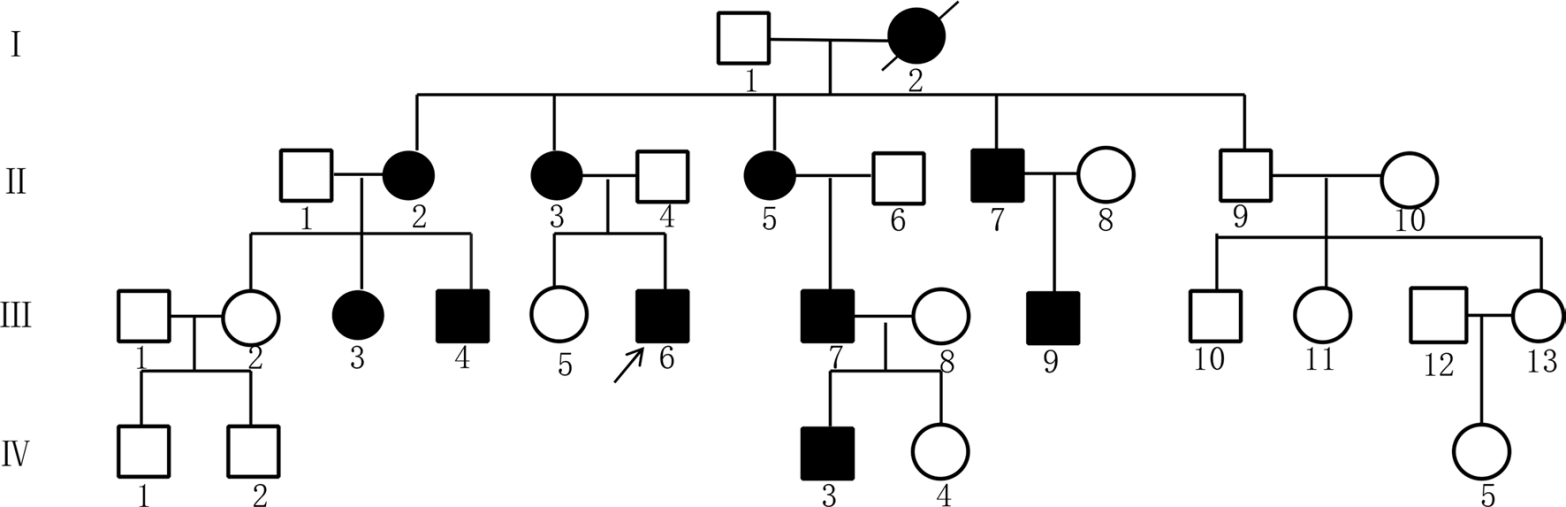
The DUH family pedigree chart. The proband is marked with an arrow. Men are represented by squares and women by circles. Black-filled symbols indicate family members with symptoms of pigmentation

**Fig. 2:**
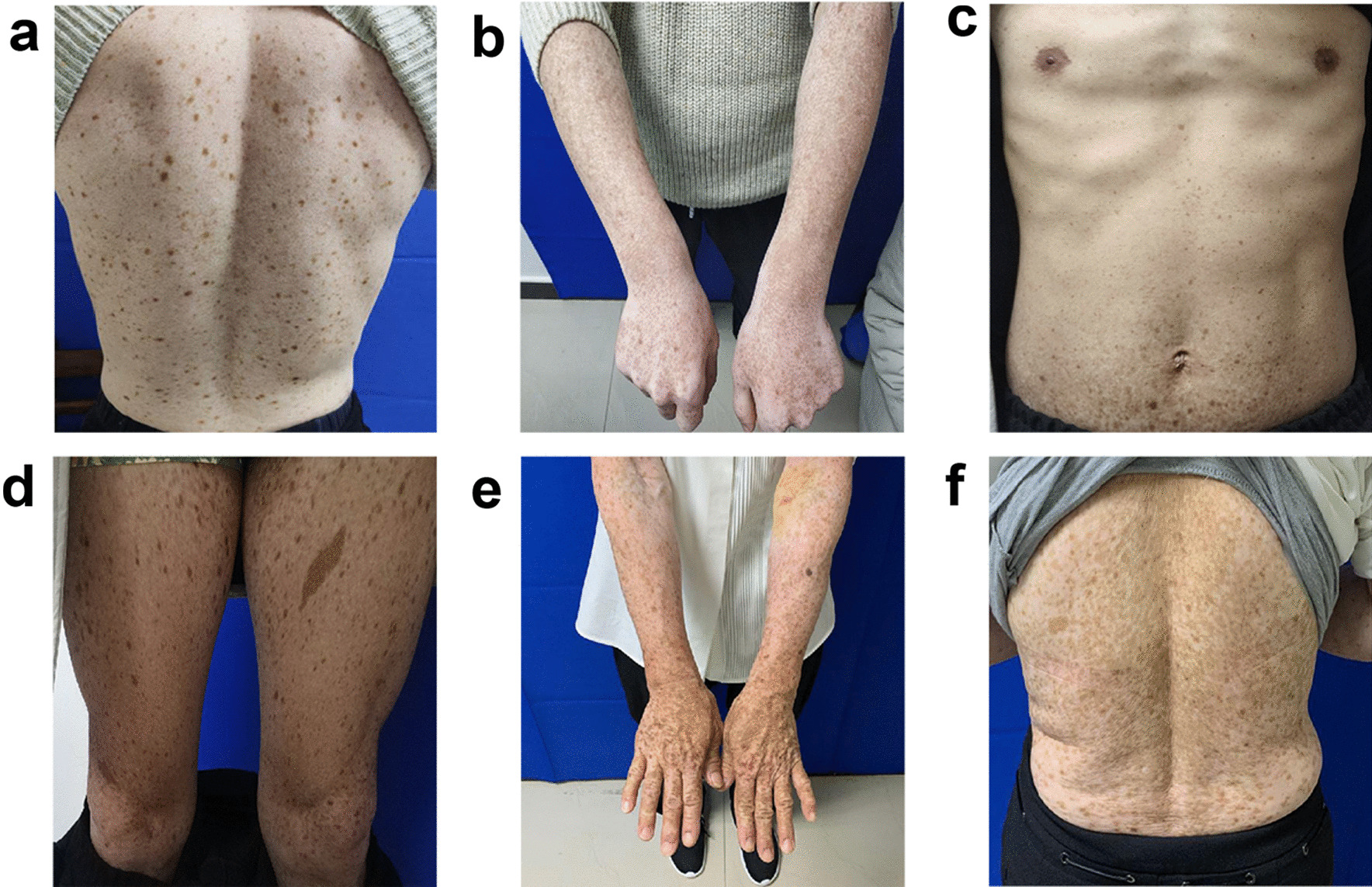
**a**, **b** Mottled hypo- and hyper-pigmented macules on the trunk and limbs in the proband. **c**, **d** Cutaneous manifestations of the proband’s cousin. Dark-brown pigmentation was diffusely distributed on his abdomen and limbs and more severely distributed on the extremities. **e**, **f** Cutaneous manifestations of the proband’s mother. Generalized hyperpigmentation spots mixed with hypopigmentation spots in a reticular pattern on their hands and back

We recruited a family with DUH with autosomal-dominant pigmentary disorder characteristics. The family pedigree is shown in Fig. 1, in which III6 was the proband. The proband was a 22-year-old male with lesions that varied in size and pigmentation throughout the body. The macules were isolated and were not associated with pain or itching; macules on both upper limbs and the back were more serious, whereas the palms, soles and mucosa were free of macules (Fig. 2a, b). The proband developed symptoms at the age of 4, with scattered spots appearing first on his face and hands and then gradually spreading to other parts of the body, such as the trunk and extremities. Macules no longer changed after approximately 15 years of age. The proband reported that the skin became slightly red after sun exposure and the skin lesions slightly worsened. Because the patient's skin colour was light, hypopigmentation spots were not obvious.

His mother (II3) and cousin (III9) underwent a similar clinical process to that of the proband, and macules spread over their whole body. Dark brown pigmentation was diffusely distributed across the proband’s cousin’s face and limbs, while the trunk showed mild symptoms (Fig. 2c, d). The lesions of the proband’s mother were more typical, with obviously generalized hyperpigmentation spots mixed with hypopigmentation spots in a reticular pattern on the hands and back (Fig. 2e, f). The proband’s sister (III5), father (II4), uncle (II9) and cousin (III11) were unaffected.

### Sanger sequencing

We collected detailed clinical data and pictures. Venous blood samples from family members II3, II4, II9, III5, III6, III9 and III11 were collected using EDTA anticoagulant tubes. DNA was extracted in accordance with the kit procedure (Sigma) and stored at − 80 °C. To identify the presence and identity of mutations in candidate genes verified by target sequencing, Sanger sequencing of polymerase chain reaction (PCR) amplicons from genomic DNA was used. Twenty-five pairs of specially designed primers were used to cover all exons of SASH1, and these primers were designed by online Primer3 software (http://bioinfo.ut.ee/primer3-0.4.0/) and are illustrated in Additional file 1. Then, the DNA samples were amplified by PCR using HotStarTaq polymerase (TAKARA). PCR products were purified by SAP (Promega) and Exo I (Epicentre) and then sequenced with a BigDye3.1 kit (ABI). The sequencing products were analysed by an ABI3730XL DNA Analyzer, and the results were analysed by PolyPhred software and confirmed by manual inspection.

## Results

**Fig. 3:**
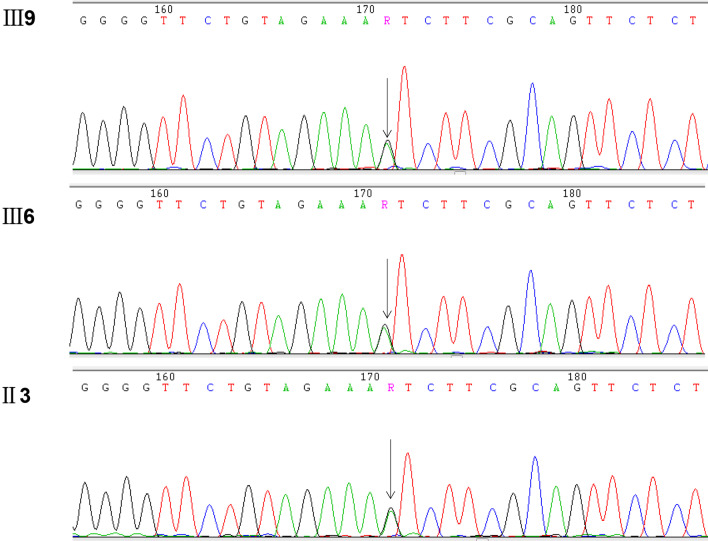
A missense mutation, c.1529G>A (p.Ser510Asn), in *SASH1* was found in the affected family members (II3, III6, and III9)

**Fig. 4:**
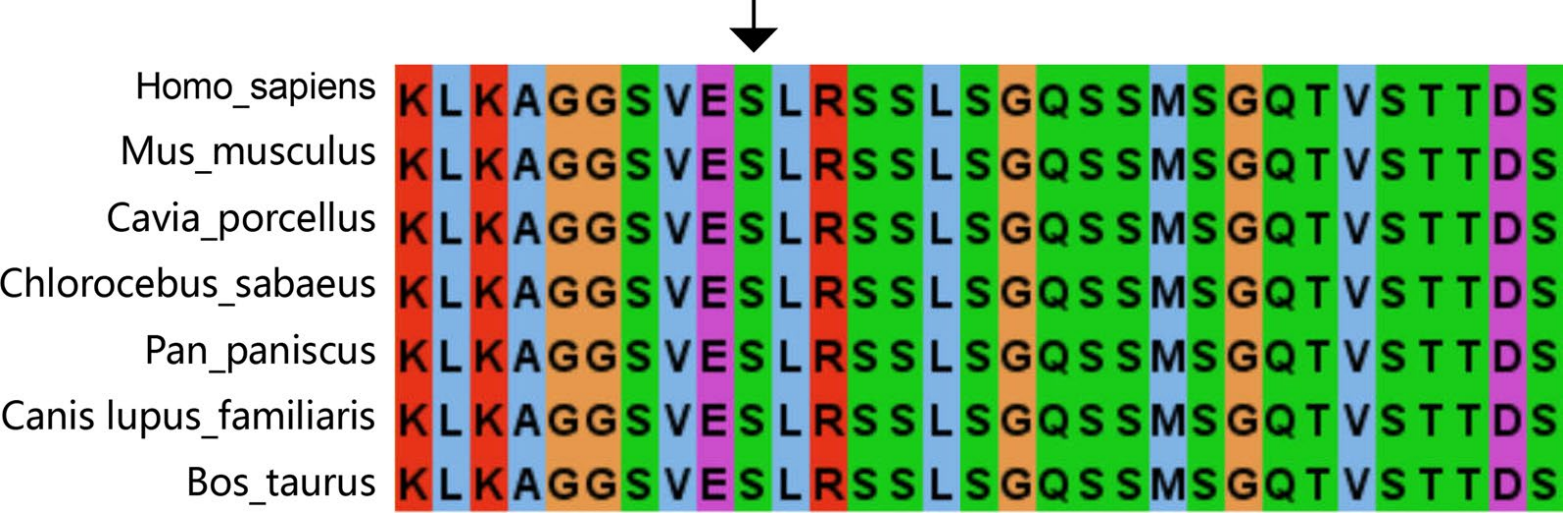
Multiple alignment of partial sequences of SASH1 from seven diverse vertebrate species performed with Clustal showing that the p.S510N (shown with an arrow) is highly conserved during evolution

According to the Sanger sequencing results, we identified a previously unreported heterozygous missense mutation [(hg19) chr6:g.148852762G>A/c.1529G>A/p.S510N] in exon 13 of the *SASH1* gene (NM_015278) in family members II3, III6, and III9 (Fig. 3), and this mutation was not present in the other four unaffected individuals (II4, II9, III5, and III11; the Sanger sequencing traces are shown in Additional file 2). This variant was the only one that cosegregated perfectly with the phenotype in this family, and the variant was not found in the National Center for Biotechnology Information Database of Short Genetic Variation (dbSNP) (https://www.ncbi.nlm.nih.gov/snp/), OMIM (https://www.omim.org/), ClinVar (https://www.ncbi.nlm.nih.gov/clinvar/), or The 1000 Genomes Project (1000G) (https://www.1000genome.org) databases. The presence of this variant resulted in an amino acid substitution from serine to asparagine (p.Ser510Asn). The p.S510N substitution occurs within the highly conserved SLY region. Moreover, all in silico predictors suggested that the observed substitution mutation is deleterious: SIFT (deleterious, score = 0.017 http://sift.bii.a-star.edu.sg), PolyPhen (probably damaging, score = 1 http://genetics.bwh.harvard.edu/pph2), and MutationTaster (disease-causing, p = 1 http://www.mutationtaster.org). Multiple sequence alignment was performed on SASH1 for seven diverse vertebral species (*Homo sapiens, Mus musculus*, *Cavia porcellus*, *Chlorocebus sabaeus*, *Pan paniscus*, *Canis lupus familiaris*, *Bos taurus*) using Clustal (https://www.ebi.ac.uk/Tools/msa/clustalo/), and the results indicated that codon 510 is highly conserved during evolution and has functional importance (Fig. 4).

## Discussion

When we reviewed the case of this family, we found a higher proportion of affected individuals in the second generation than expected for a heterozygous mutation. Therefore, we hypothesize that the mutation carried by the proband's grandmother may be homozygous, which means that II9 may also carry the pathogenic mutation. The fact that no features of DUH were observed in II9 (Additional file 3) may be related to reduced penetrance, which is not uncommon in genetic disease and often explains why individuals with disease-causing mutations fail to express the phenotype of the disease [6]. The patient reported that he was not breastfed because his mother died shortly after giving birth to him, which may be the reason for incomplete penetrance. We conducted Sanger sequencing to verify whether the possible pathogenic variant was present in II9, but as shown in the sequencing trace, II9 did not carry the mutation. Therefore, the causes of the unusual prevalence of affected individuals in the second generation remain poorly defined.

The *SASH1* gene, which is located at 6q2e4.3, contains an SLY domain, an SH3 domain, and two SAM domains. The *SASH1* gene was originally thought to work as a tumour suppressor for breast cancer [7] and colon cancers [8] and has been demonstrated to have a crucial regulatory role in tumorigenesis. Subsequently, the role of *SASH1* in pigmentation disorders has been continuously explored, as it participates in the DUH process by modulating melanocyte transepithelial migration through the Gas-SASH1-IQGAP1-E-cadherin signalling pathway [5] or, with the help of novel p53/α-MSH/POMC/Gαs/SASH1 crosstalk, p53 regulates the ERK1/2/CREB cascade and then causes hyperpigmentation [9]. Recently, in vivo studies on *SASH1* function were investigated in a heterozygous mouse model in which the *SASH1* c.1654T>G (p.Tyr551Asp, Y551D) mutation was knocked in. Xu et al. pointed out that *SASH1* regulates the expression of Mitf in the nucleus by acting as a scaffold molecule that participates in the assembly of the SASH1-MITF molecular complex and promotes the hyperpigmentation phenotype in the pathogenesis of DUH and other dermatoses related to abnormal pigmentation [10].

**Fig. 5:**
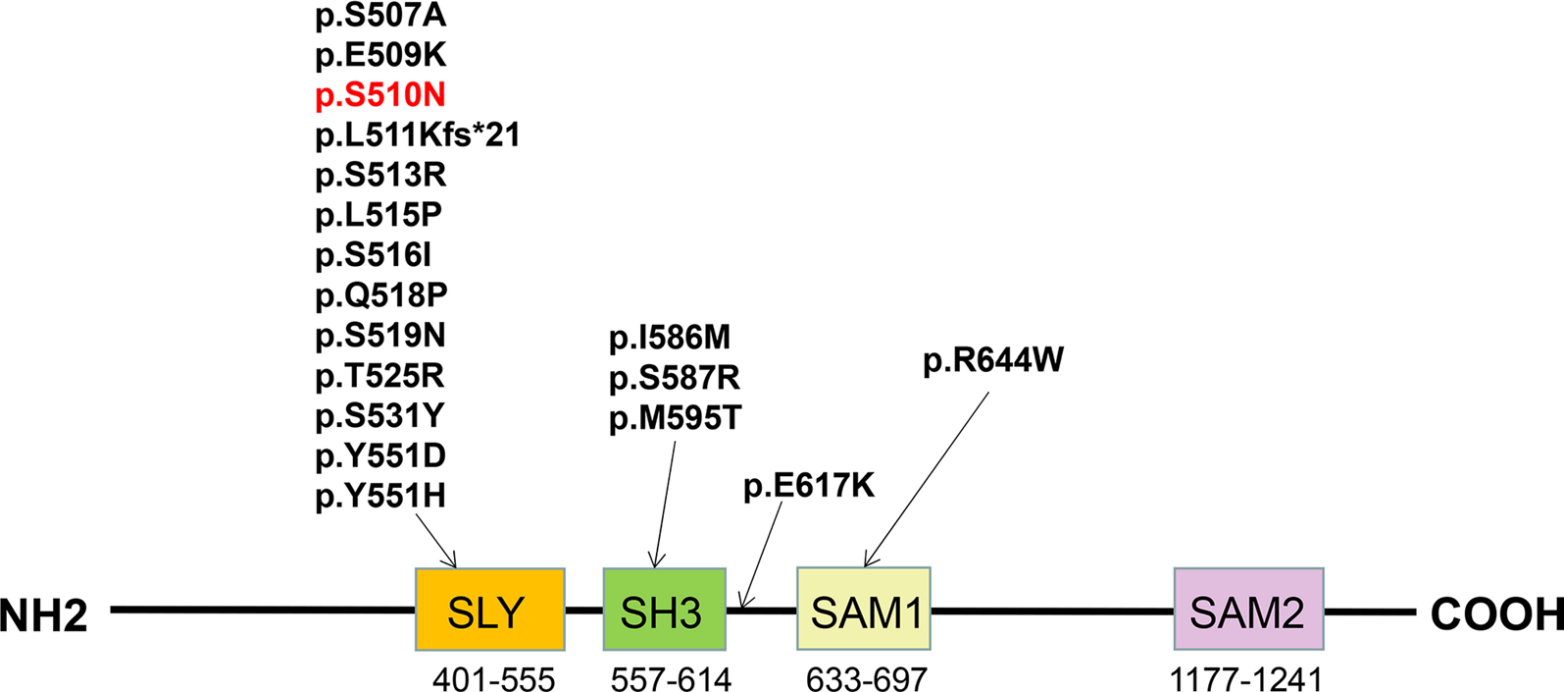
The distribution of all mutations inducing pigmentary anomalies in the protein domain of *SASH1*

To date, 17 heterogeneous missense mutations in *SASH1* have been confirmed to be associated with pigmentation disorders, among which eight mutations result in DUH [5, 11, 12] and nine are responsible for multiple lentiginous phenotypes [13–16]; furthermore, the missense mutation c.1849G>A was reported to result in a loss of pigmentation with palmoplantar keratoderma and skin carcinoma [17]. The distribution of all mutations in the protein domain of *SASH1* inducing pigmentary anomalies is shown in Fig. 5, and the information related to the mutations is shown in Table 1. Most mutations are located in the SLY domain, which is highly conserved and seen as a hotspot mutation region [13]. Zhou et al. [5] first identified three missense mutations in *SASH1*, c.2126T>G (p.Tyr551Asp), c.2019T>C (p.Leu515Pro), c.2000G>A (p.Glu509Lys). In 2016, Chinese scholars recruited a family with nine affected members who manifested irregular light-brown- to dark-brown-pigmented spots over the whole body; the remaining skin was uniformly hypopigmented, and a fourth missense mutation, c.1761C>G (p.Ser587Arg), was identified. Moreover, Zhong et al. [11] reported two unrelated DUH pedigrees with novel missense mutations in *SASH1*. Proband 1 was a 25-year-old female born with normal skin pigmentation. At the age of 3, the freckle-like macules gradually spread to her trunk, face, neck and limbs, with accentuation on sun-exposed areas intermingled with hypopigmented spots. Proband 2 was a 42-year-old male who shared similar clinical manifestations with an earlier onset time. Analysis revealed the presence of the c.1784T>C (p.M595T) and c.1651T>C (p.Y551H) missense mutations in the *SASH1* gene. The most recent case was described by Wu et al. [12], who described the case of a 6-year-old girl with generalized hyperpigmented spots, and her family members shared similar symptoms. After genetic analysis, a novel missense mutation, c.1553A>C, was discovered.

**Table 1:** *SASH1* mutations associated with pigmentation disorders

Number	Gene	Clinical phenotypes	Mode of inheritance	Onset age	Nucleotide change	Amino acid change	References
1	*SASH1*	Dyschromatosis universalis hereditaria	–	–	c.2000G>A	p.Glu509Lys	Zhou et al. [5]
2	*SASH1*	Dyschromatosis universalis hereditaria	–	–	c.2019T>C	p.Leu515Pro	Zhou et al. [5]
3	*SASH1*	Dyschromatosis universalis hereditaria	–	–	c.2126T>G	p.Tyr551Asp	Zhou et al. [5]
4	*SASH1*	Dyschromatosis universalis hereditaria	Autosomal-dominant inheritance	< 1 year	c.1761C>G	p.Ser587Arg	Chinese Journal of Dermatology
5	*SASH1*	Dyschromatosis universalis hereditaria	Autosomal-dominant inheritance	3 years	c.1784T>C	p.Met595Thr	Zhong et al. [11]
6	*SASH1*	Dyschromatosis universalis hereditaria	Autosomal-dominant inheritance	7 months	c.1651T>C	p.Tyr551His	Zhong et al. [11]
7	*SASH1*	Dyschromatosis universalis hereditaria	Autosomal-dominant inheritance	2 years	c.1553A>C	p.Gln518Pro	Wu et al. [12]
8	*SASH1*	Dyschromatosis universalis hereditaria	Autosomal-dominant inheritance	4 years	c.1529G>A	p.Ser510Asn	Our present study
9	*SASH1*	Lentiginous phenotype	Autosomal-dominant inheritance	1 year	c.1556G>A	p.Ser519Asn	Shellman et al. [13]
10	*SASH1*	Lentiginous phenotype	Autosomal-dominant inheritance	3 years	c.1537A>C	p.Ser513Arg	Zhang et al. [14]
11	*SASH1*	Lentiginous phenotype	Sporadic	–	c.1527_1530dupAAGT	p.Leu511Lysfs*21	Zhang et al. [14]
12	*SASH1*	Lentiginous phenotype	Autosomal-dominant inheritance	18 months	c.1519T>G	p.Ser507Ala	Wang et al. [15]
13	*SASH1*	Lentiginous phenotype	Sporadic	14 months	c.1758C>G	p.Ile586Met	Yuta Araki et al. [16]
14	*SASH1*	Lentiginous phenotype	Autosomal-dominant inheritance	2 years	c.1592C>A	p.Ser531Tyr	Yuta Araki et al. [16]
15	*SASH1*	Lentiginous phenotype	Autosomal-dominant inheritance	3 years	c.1930C>T	p.Arg644Trp	Yuta Araki et al. [16]
16	*SASH1*	Lentiginous phenotype	Sporadic	2 years	c.1574C>G	p.Thr525Arg	Yuta Araki et al. [16]
17	*SASH1*	Lentiginous phenotype	Sporadic	8 months	c.1547G>T	p.Ser516Ile	Yuta Araki et al. [16]
18	*SASH1*	Dyschromatosis-universalis-hereditarian-like pigmentation	Autosomal-recessive inheritance	1 year	c.1849G>A	p.Glu617Lys	Courcet et al. [17]

In addition to DUH, nine missense mutations in *SASH1* (p.S513R, p.L511Kfs*21, p.S519N, p.S507A, p.I586M, p.S531Y, p.R644W, p.T525R and p.S516I) were reported to be associated with the lentiginous phenotype. Multiple lentigines are known to be characteristic of generalized lentiginosis, which is an autosomal-dominant genetic disease with abnormal pigmentation that can be associated with cardiovascular diseases, mental retardation, neurological deafness and other abnormalities. The common pathogenic genes reported thus far include *PTPN11, BRAF* and *RAF1* [18–20]; therefore, whether these two diseases have overlapping virulence genes or, due to similar phenotypes, these cases were misdiagnosed are worth further discussion.

*ABCB6* was first determined to be the causative gene of DUH by Zhang et al. when genome-wide linkage analysis and exome sequencing was completed; c.1067T>C (p.Leu356Pro), c.508A>G (p.Ser170Gly) and c.1736G>A (p.Gly579Glu) were identified in the DUH family or sporadic cases [4]. To date, nine mutations have been discovered in *ABCB6* associated with DUH. The other six mutations were c.1663C>A (p.Gln555Lys), c.459delC (p.Trp154Glyfs*96), c.1358C>T (p.Ala453Val), c.964A>C (p.Ser322Lys), c.1270T>C (p.Tyr424His), and c.2017A>G (p.Thr673Ala) [21–25]. Furthermore, a novel missense mutation in *ABCB6* (p.N467S) was reported in a case of xeroderma pigmentosum C combined with DUH-like pigmentation [26]. Wu et al. [12] pointed out that, compared with the disease caused by *SASH1* mutation, the disease caused by *ABCB6* mutation showed more typical manifestations, such as generalized mottled hyperpigmented macules mixed with hypopigmented macules arranged in a reticular pattern. In contrast, diseases caused by *SASH1* mutations are more likely to be confused with generalized lentiginosis. We have different views on diseases caused by *SASH1* mutations, and we present case examples of our views. As shown above, although the mutation in the proband was the same as that in his mother and cousin, their clinical manifestations were different. Possibly due to the light colour of his skin, the proband had milder hypopigmentation than his mother and cousin. We hypothesized that because clinical manifestations are influenced by skin colour, age, and other environmental factors, such as ultraviolet (UV) exposure, the correlation of genotype and phenotype in patients with DUH needs to be further investigated.

## Conclusions

In conclusion, we found a novel mutation, c.1529G>A of *SASH1* in a family with DUH. This novel mutation broadens the mutational spectrum of this gene. As further research on the genetics of DUH is carried out and more pathogenic genes and mutation sites are discovered, a further understanding of the genetic characteristics of DUH will be obtained, providing the basis for the clinical implementation of gene therapy for DUH.

## Supplementary Information

**Additional file 1.** The primer of *SASH1* gene.

**Additional file 2.** Cutaneous manifestation of the proband’s uncle (II9).

**Additional file 3.** The Sanger sequencing trace of unaffected individuals (II4, II9, III5, III11).

## Data Availability

The datasets analyzed during the present study are available from Zenodo (number: 4898895, https://zenodo.org/record/4898895#.YLnm1Mij7RQ). The novel variant has been submitted to ClinVar database (accession number: SCV001548229, https://www.ncbi.nlm.nih.gov/clinvar/variation/1048561/). The relevant datasets links were as follows: dbSNP (https://www.ncbi.nlm.nih.gov/snp/), OMIM (https://www.omim.org/), ClinVar (https://www.ncbi.nlm.nih.gov/clinvar/), and 1000G (http://www.1000genomes.org/).

## Abbreviations

DUH: Dyschromatosis universalis hereditaria
EDTA: Ethylenediaminetetraacetic acid
PCR: Polymerase chain reaction
dbSNP: National Center for Biotechnology Information Database of Short Genetic Variation
OMIM: Online Mendelian Inheritance in Man
1000G: The 1000 Genomes Project
SIFT: Scale invariant feature transform
UV: Ultraviolet
