# Supplementary figures and images for: Novel missense mutation of SASH1 in a Chinese family with dyschromatosis universalis hereditaria
Source: BMC Med Genomics. 2021 Jun 26;14:168. doi: 10.1186/s12920-021-01014-w (PMC8236144; doi:10.1186/s12920-021-01014-w)

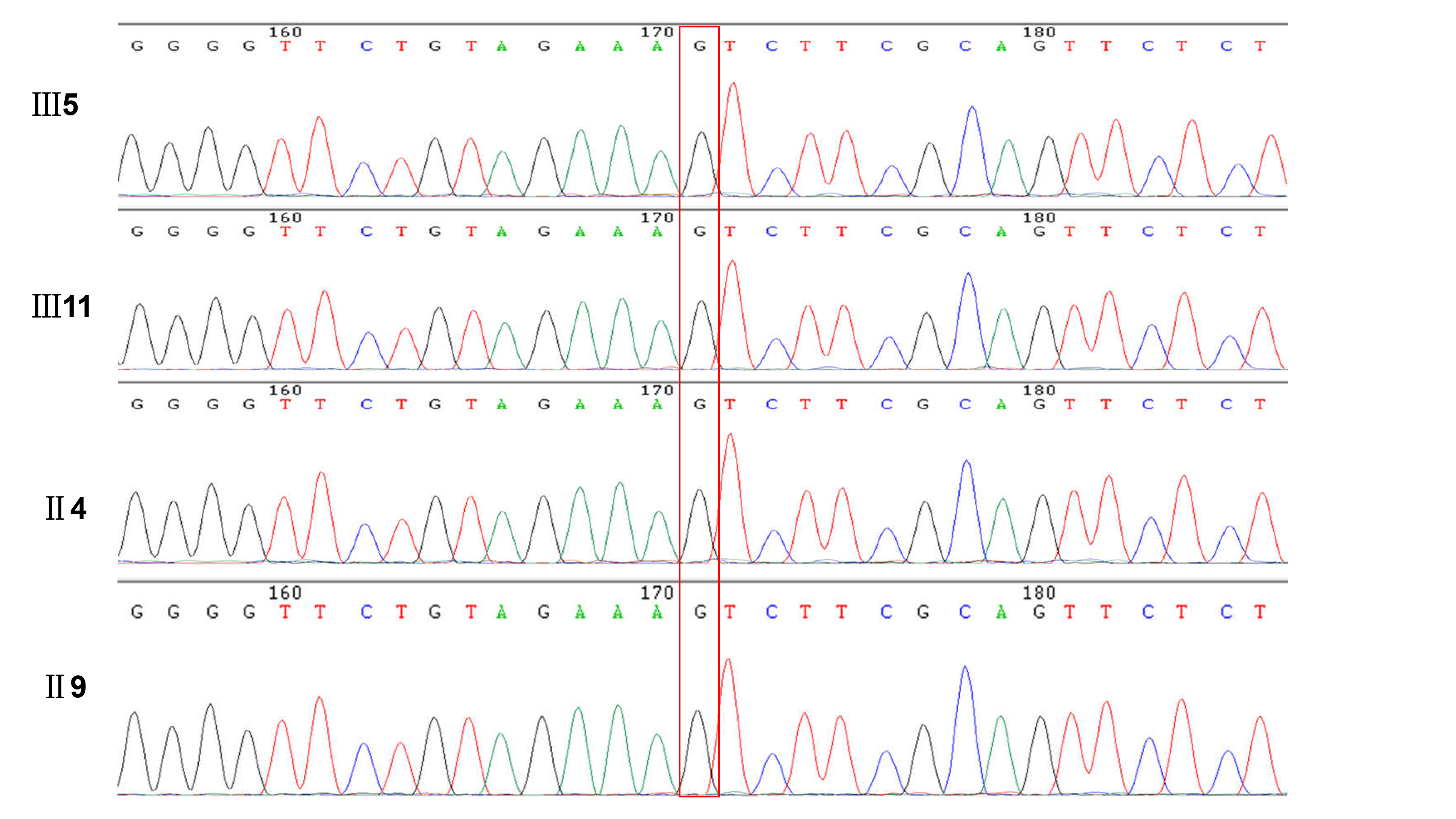

Supplement: Supplementary file 2 — Additional file 2. Cutaneous manifestation of the proband’s uncle (II9). [file 12920_2021_1014_MOESM2_ESM.tif]

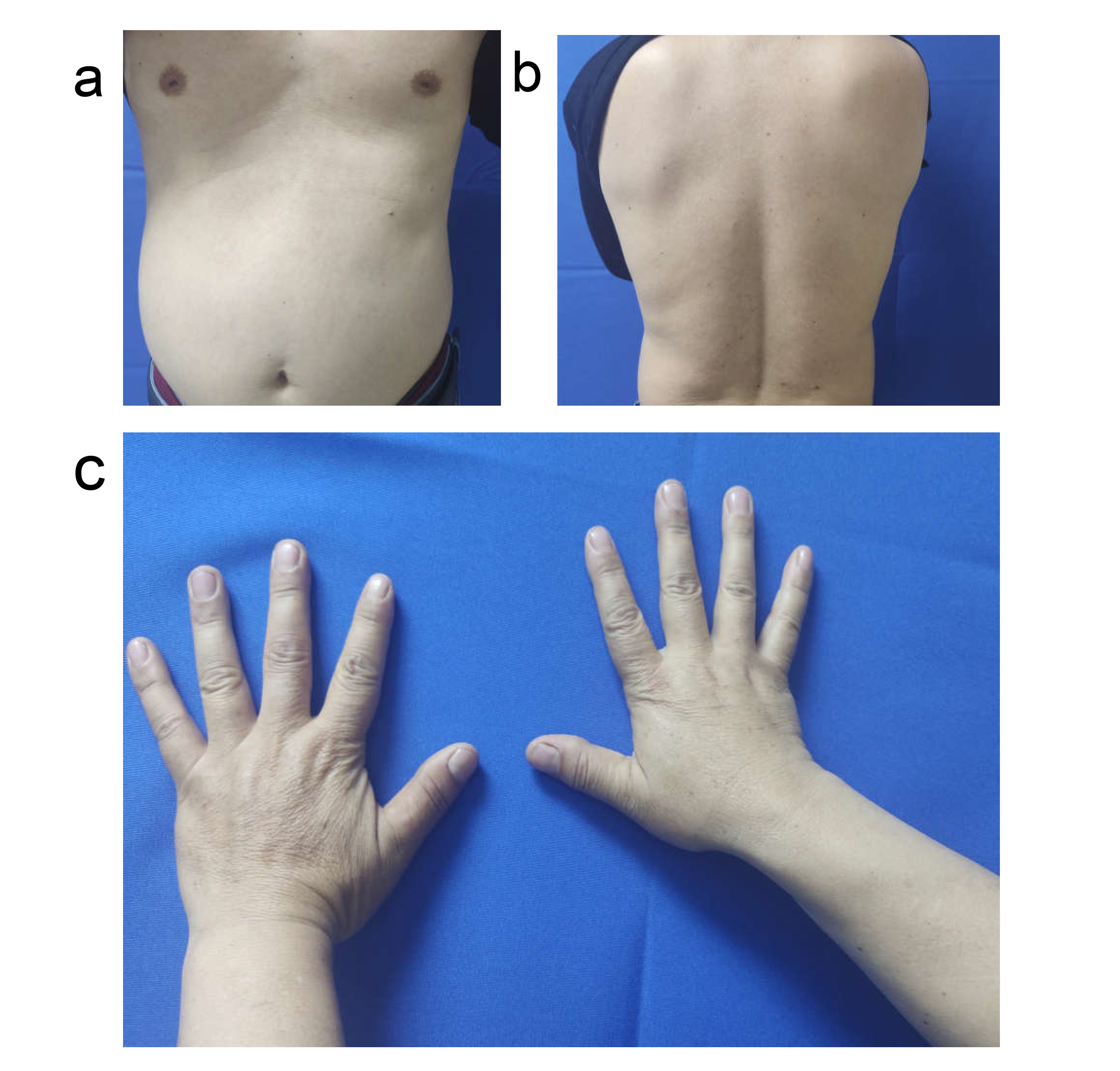

Supplement: Supplementary file 3 — Additional file 3. The Sanger sequencing trace of unaffected individuals (II4, II9, III5, III11). [file 12920_2021_1014_MOESM3_ESM.tif]
